# Cu/ZrO_2_ Catalyst Modified with Y_2_O_3_ for Effective and Stable Dehydration of Glycerol to Acetol

**DOI:** 10.3390/molecules29020356

**Published:** 2024-01-11

**Authors:** Zhoubing Liang, Huan Li, Jianrong Xie, Songshou Ye, Jinbao Zheng, Nuowei Zhang

**Affiliations:** Department of Chemical and Biochemical Engineering, National Engineering Laboratory for Green Productions of Alcohols-Ethers-Esters, College of Chemistry and Chemical Engineering, Xiamen University, Xiamen 361005, China15059152023@163.com (H.L.); jianronx@xmu.edu.cn (J.X.); clow4648@163.com (S.Y.); jbzheng@xmu.edu.cn (J.Z.)

**Keywords:** glycerol, dehydration, acetol, acidity, Y_2_O_3_ modification

## Abstract

Glycerol is a main by-product of biodiesel production, and its further processing is essential for the biorefinery. In this paper, a highly active and stable catalyst for the catalytic dehydration of glycerol to acetol is obtained by modifying a Cu-Zr (ZrO_2_ supported Cu) catalyst with Y_2_O_3_ using a co-precipitation method. It is found that the addition of Y_2_O_3_ effectively enhances the catalytic performance of Cu-Zr. Cu-Zr reaches the highest selectivity (82.4%) to acetol at 24 h. However, the selectivity decreases to 70.1% at 36 h. The conversion also decreases from 99.2 to 91.1%. Cu-Zr-Y exhibits very high activity and very good stability. During a 250 h reaction, no deactivation is observed, and the conversion and selectivity remains ~100% and ~85%, respectively. The catalysts are characterized by XRD, TEM, H_2_-TPR, and NH_3_-TPD. The results reveal that Y_2_O_3_ not only improves the dispersion of Cu and the acidity of the catalyst but also restrains the agglomeration of Cu particles and assists retaining the main structure of support under reaction conditions. The high dispersion, high acidity content, and stable structure contributes to the excellent catalytic performance of Cu-Zr-Y.

## 1. Introduction

Over the last few decades, determining how to valorize glycerol has been widely investigating since the steep increasing biodiesel production results in an excess supply of glycerol [1]. Glycerol is a highly reactive molecule as it possesses three hydroxyl functional groups. Based on the heterogeneous catalysis, glycerol can be converted into many high-added-value products, including selective oxidation to dihydroxyacetone, glyceric acid, hydroxypyruvic acid [2], mesooxalic acid [3], tartronic acid or lactic acid [4], hydrogenolysis to propanediols [5] or ethylene glycol [6], catalytic dehydration to acrolein or acetol [1], etc. Among the derivative production, catalytic dehydration to acetol has been receiving great attention due to the broad use of acetol as a chemical platform [7]. Acetol can be used to produce propylene glycol, propionaldehyde, acetaldehyde, and furan derivatives. Acetol is also used in cosmetics and pharmaceutical industries.

A catalyst plays a very important role for the catalytic dehydration of glycerol to acetol. Both noble metal-based (Ru, Pt, Pd) and transition metal-based (Ni, Co, Cu) catalysts have been studied. Over Ru-, Pt-, Pd-based catalysts, glycerol is more easily converted into 1,2-propanediol since noble metals are potent activators of hydrogen molecules [8,9,10]. For Ni- and Co-based catalysts, they are active for breaking C-C bonds of a glycerol molecule and thus results in low selectivity to acetol [11,12,13,14]. The Cu-based catalysts exhibited promising catalytic performance for the dehydration of glycerol to acetol due to the high reactivity for breaking C-O bonds but low efficiency for the cleavage of C-C bonds [15,16,17,18,19,20].

CuO/SiO_2_ catalysts were prepared and investigated for the dehydration of glycerol to acetol. It was found that increasing the copper oxide dispersion increased the acidity of the catalyst and thus promoted the catalytic performance for dehydrate glycerol selectively to acetol. Both the number of exposed Cu active sites and the acidity generated in the CuO nanoparticles contributed to the developed catalytic performance [21]. Cu-Ba, Cu-Mg, Cu-Zr, Cu-Zn, and Cu-Al catalysts were prepared by co-precipitation method and tested for the dehydration of glycerol to acetol. The results indicated that the Cu-Mg, Cu-Zr, and Cu-Al, having higher acid strength and predominant Brønsted acidity, gave the highest acetol selectivity, while the Cu-Zn with lower acidity showed very poor selectivity to acetol. It was also found that the catalysts containing metallic Cu exhibited higher catalytic activity and acetol selectivity [22]. The catalytic performance of calcium hydroxyapatites (HAP) containing cobalt or copper catalysts were comparatively studied for glycerol conversion to acetol production. Copper-containing catalysts were more active than cobalt catalysts since the former exhibited a stable libethenite phase and contained great acidity [23]. γ-Al_2_O_3_ and TiO_2_ supported copper and nickel catalysts were prepared for conversion of glycerol to hydroxyacetone. Among the studied catalysts, Cu/γ-Al_2_O_3_ and Cu/TiO_2_ gave higher glycerol conversion and selectivity to hydroxyacetone. It was also found that Cu/γ-Al_2_O_3_ suffered deactivation due to the carbonaceous deposits [24]. Braga et al. studied the effect of the support acidity and the copper surface characteristics on the catalytic performance for the gas-phase conversion of glycerol to acetol by using mesoporous mixed copper-aluminum oxides and copper-silicon oxides as catalysts. The results showed a clear dependence of the glycerol conversion to acetol with the Cu metal surface, and the initial catalytic properties did not depend on the support acidity. However, the support acidity strongly influenced the catalyst stability. The Cu-Al solid deactivated continuously due to the higher acidity and the greater carbon [25]. Batiot-Dupeyrat et al. reported that Cu-MgF_2_ was much more active than Cu-MgF(OH) and Cu-MgO for the reaction of glycerol dehydration to hydroxyacetone. The reason for the best catalytic performance was that Cu-MgF_2_ possessed the higher amount of Lewis acid sites and can stabilize copper at the +1-oxidation state [26]. The highest yield of acetol (~80%) from glycerol was obtained over a copper chromite catalyst in a reactive distillation system [27]. However, such a process contained the main disadvantage, namely that copper chromite catalyst is toxic, and the distillation system is difficult to be scaled-up.

Up to now, acetol is only produced as an intermediate in some companies but yet not manufactured as a single chemical from glycerol. The main reason is that the catalytic performance of the catalyst, especially for the selectivity and stability, cannot meet the requirement of the industrial applications. For Cr-free catalysts, both Cu/Al_2_O_3_ and Cu/ZrO_2_ are promising due to the high selectivity to acetol [19,22]. Although Cu/Al_2_O_3_ is more active than Cu/ZrO_2_, it suffered from deactivation due to the fact that the strong acidity of Al_2_O_3_ resulted in the formation of carbonaceous deposits [24,25]. Compared with Al_2_O_3_, ZrO_2_ possesses a weaker acidity, which maybe reduce the formation of carbonaceous deposits. In this paper, ZrO_2_-supported Cu material (Cu-Zr) was selected as a catalyst for the dehydration of glycerol to acetol. Both the Cu active sites and acidity property determines the catalytic performance. Our previous results indicated that Y_2_O_3_ can stabilize ZrO_2_ support, suppress the transformation from the amorphous phase to crystal ZrO_2_, and ensure Ni dispersed well [28]. The ZrO_2_ support with the amorphous phase made the Cu/ZrO_2_ catalyst possess a better Cu dispersion [29]. Therefore, Y_2_O_3_ was selected as a promoter and introduced into Cu-Zr to improve the Cu dispersion, increase the acidity, and stabilize the ZrO_2_ support. The results indicated that Cu-Zr-Y was highly active, selective, and stable for the dehydration of glycerol to acetol. Characterizations including XRD, H_2_-TPR, NH_3_-TPD, BET, XPS, SEM, and TEM were performed to study the relationship between the catalytic performance and physicochemical properties.

## 2. Results and Discussion

**Figure 1:**
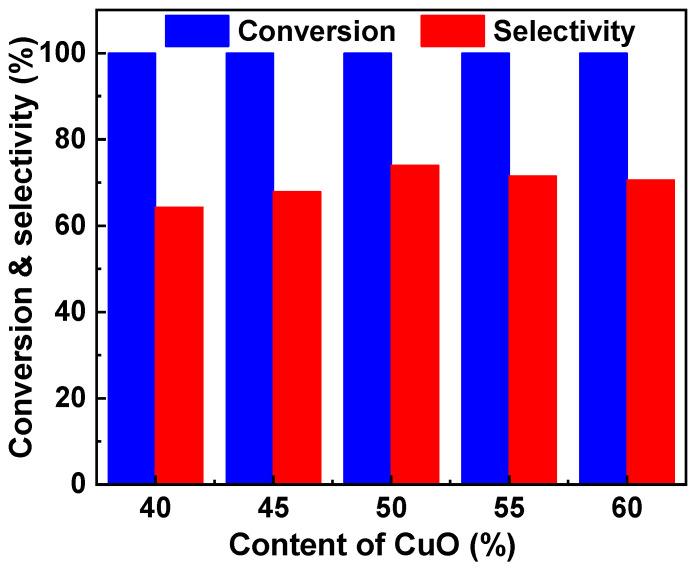
The effect of CuO content on the activity and selectivity of the Cu-Zr catalyst. Conditions: temperature = 220 °C, time = 24 h, LHSV = 0.45 h^−1^.

The number of Cu active sites is one of the two factors that mainly determine the catalytic performance for the dehydration of glycerol to acetol. It was reported that the catalyst with ~50% CuO exhibited the best catalytic performance [18]. Therefore, the effect of CuO content in the range from 40 to 60% on the catalytic performance of the Cu-Zr catalyst was first investigated, and the results are shown in Figure 1. The five catalysts exhibited high activity, and all the conversions of glycerol were higher than 99%. However, the CuO content affected the selectivity of acetol to a great extent. The Cu-Zr with 40% CuO showed a low selectivity to acetol, and the value was only 64.3%. Increasing the CuO content can improve the selectivity to acetol. The Cu-Zr with 50% CuO showed the highest selectivity (74.0%) to acetol. When further increasing the CuO content, the selectivity decreased. When the CuO content increased to 60%, the selectivity decreased to 70.6%.

**Figure 2:**
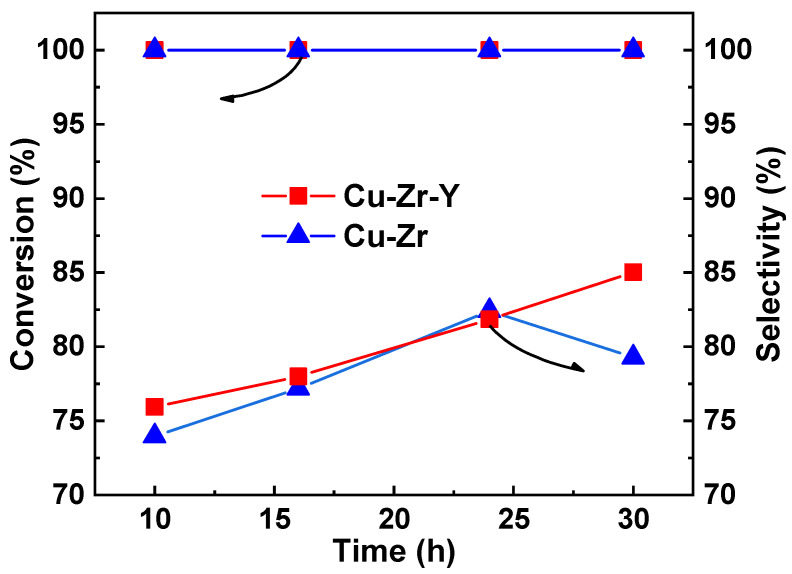
The effect of Y_2_O_3_ on the catalytic performance of the Cu-Zr catalyst. Conditions: temperature = 220 °C, LHSV = 0.45 h^−1^.

Increasing Cu dispersion can increase Cu active sites and the acidity of the catalyst, which was favorable for the formation of acetol from glycerol. Y_2_O_3_ was introduced into the Cu-Zr catalyst to improve Cu dispersion, acidity property, and the selectivity to acetol. Figure 2 shows the activity and selectivity of Cu-Zr and Cu-Zr-Y during a 30 h reaction. It can be seen that the initial catalytic performance of the two catalysts was very closed. The conversion of glycerol was higher than 99%, and the selectivity of acetol was around 75%. With rection proceeding, the high conversion remained unchanged while the selectivity increased. At 24 h, the selectivity of the two catalysts increased to around 82%. However, with a further increasing reaction time, the selectivity of the two catalysts exhibited different changing trends. After the 30 h reaction, the selectivity of Cu-Zr-Y further increased to 85.0% while that of Cu-Zr decreased to 79.3%. Clearly, the introduction of Y_2_O_3_ can effectively improve the catalytic performance of Cu-Zr.

The effect of CuO content in the range of 45 to 60% on the catalytic performance of Cu-Zr-Y catalysts was investigated. As shown in Table 1, the CuO content showed little effect on the activity, while it affected the selectivity to some extent. The Cu-Zr-Y catalyst with 45% CuO present the lowest selectivity (81.7%). When the CuO content was increased from 45 to 50%, the selectivity was increased from 81.7 to 85.0%. However, with further increasing the CuO content, the selectivity decreased gradually. When the CuO content was 55 and 60%, the selectivity was 84.3 and 83.1%, respectively. Except for acetol, the other products were also determined. Different with acetol, all the catalysts gave the very closed selectivity to 1,2-propanediol (~7%), and there were a small number of other products that were detected, including propionic acid, acetic acid, acetaldehyde, ethylene glycon.

Compared with Cu oxide, metallic Cu is much more active and selective for the dehydration of glycerol to acetol. Therefore, the effect of reduction temperatures on the catalytic performance of Cu-Zr-Y was studied in the range of 250 to 300 °C, and the results are shown in Table 2. It can be seen that the three catalysts reduced at different temperatures gave the same high activity. However, a big difference was observed for their selectivity. The catalyst reduced at 300 °C presented the highest selectivity. The selectivity of the two catalysts reduced at 250 and 350 °C was 78.5 and 82.7%, respectively, while that for the catalyst reduced at 300 °C was 85.4%. Clearly, 300 °C was the preferred reduction temperature.

**Figure 3:**
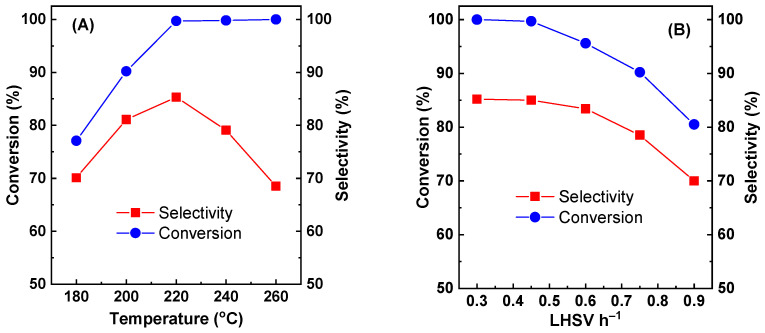
The effect of temperature (**A**) and LHSV (**B**) on the catalytic performance of Cu-Zr-Y.

Figure 3A shows the effect of the reaction temperature on the glycerol conversion and acetol selectivity of Cu-Zr-Y with 50% CuO loading. The reaction time was 30 h. The LHSV was 0.45 h^−1^. The reaction temperature strongly affected the catalytic performance. At 180 °C, Cu-Zr-Y exhibited poorer activity and selectivity. The conversion and selectivity were only 77.1 and 70.1%, respectively. With increasing reaction temperature, both conversion and selectivity increased sharply. At 220 °C, conversion and selectivity reached the maximum. The conversion was 99.7%, and the selectivity was 85.0%. With a further increasing reaction temperature, the high conversion remained unchanged, while the selectivity dropped drastically. At 260 °C, the selectivity was decreased to 68.5%. The possible reason was that the high temperature favored C-C bond cleavage instead of dehydration.

The effect of the LHSV on the catalytic performance of Cu-Zr-Y with 50% CuO loading is shown in Figure 3B. The reaction temperature was 220 °C. The reaction time was 30 h. At an LHSV below 0.45 h^−1^, the catalyst exhibited both high activity and high selectivity. The conversion was as high as ~100%, and the selectivity was higher than 85%. When the LHSV was above 0.45 h^−1^, both conversion and selectivity decreased with the LHSV increase. When the LHSV was increased from 0.45 to 0.90, the conversion and selectivity was decreased from 99.7 and 85.1% to 85.0 and 70.0%, respectively. At a low LHSV, there was enough active sites to ensure glycerol converted to acetol. Therefore, the catalyst presented excellent catalytic performance. At a high LHSV, glycerol cannot be converted in time and intermediates cannot be effectively converted to acetol. Therefore, both conversion and selectivity decreased with the LHSV increase.

**Figure 4:**
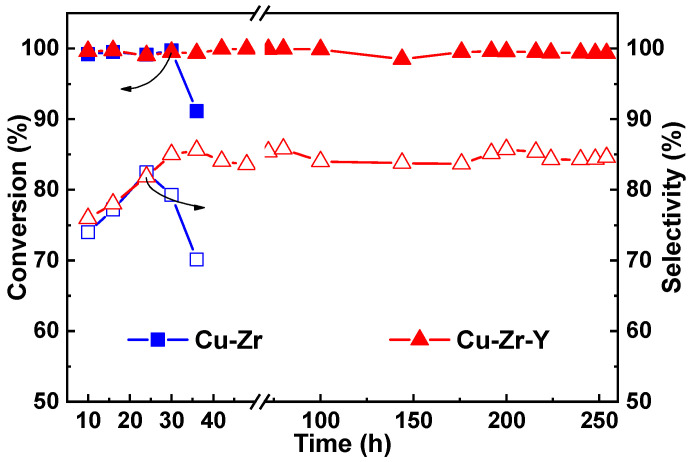
The stability of the Cu-Zr and Cu-Zr-Y catalyst. Conditions: temperature = 220 °C, LHSV = 0.45 h^−1^.

The stability of the catalyst is one of the most important parameters in the practical application. The stability of Cu-Zr and Cu-Zr-Y with 50% CuO loading was tested, and the results are shown in Figure 4. As can be seen, Cu-Zr had a poor stability. After the 36 h reaction, the conversion and selectivity were decreased from 99.2 and 82.4% to 91.1 and 70.1%, respectively. The Cu-Zr-Y catalyst provided not only high activity but also good stability. It showed the high conversion of glycerol (~100%) and high selectivity to acetol (~85%) without any loss in activity and selectivity even after the 250 h catalytic reaction. This observation further indicated that the Cu-Zr-Y catalysts were superior in catalytic performance for the reaction of the dehydration of glycerol to acetol to the Cu-Zr catalysts.

**Figure 5:**
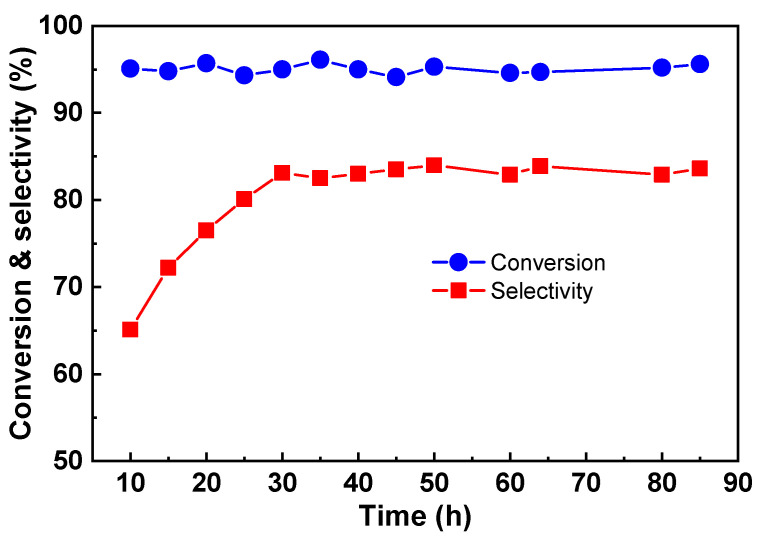
The stability of the Cu-Zr-Y catalyst. Conditions: temperature = 220 °C, LHSV = 0.60 h^−1^.

During 250 h catalytic reaction, the conversion of Cu-Zr-Y was higher than 99%. In this case, deactivation may occur, but it is not visible. To further test the stability of Cu-Zr-Y, the catalytic performance was investigated at LHSV = 0.60 h^−1^, and the result is shown in Figure 5. When the LHSV was increased from 0.45 to 0.60 h^−1^, both conversion and selectivity decreased to some extent. After the 30 h reaction, the conversion and selectivity were 95.0 and 83.1%, respectively. This observation indicated that, at LHSV = 0.60 h^−1^, the catalyst cannot offer enough active sites to convert glycerol. In spite of the fact that glycerol was not completely converted, Cu-Zr-Y still exhibited good stability. Even after the 85 h reaction, the conversion and selectivity remained unchanged, and no loss in activity and selectivity was observed. This result further confirmed the excellent stability of the Cu-Zr-Y catalyst.

**Figure 6:**
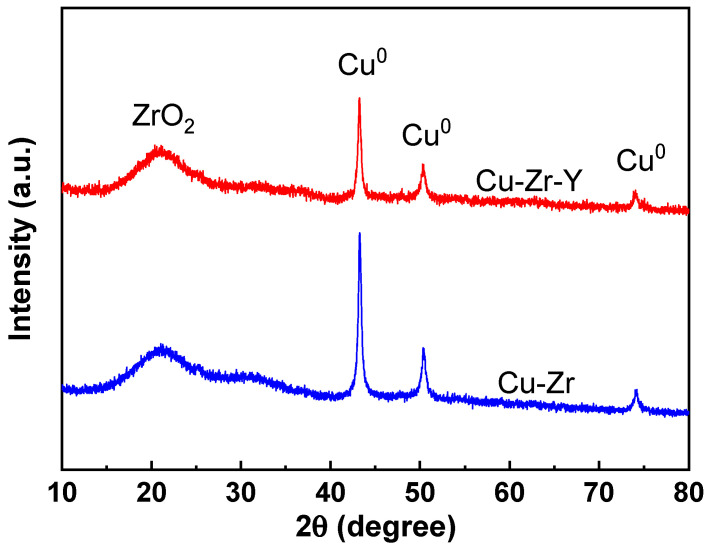
The XRD patterns of the reduced Cu-Zr and Cu-Zr-Y catalysts.

To investigate the promotional effect of Y_2_O_3_, both the Cu-Zr and the Cu-Zr-Y catalyst were characterized. Figure 6 shows the XRD patterns of Cu-Zr and Cu-Zr-Y reduced by H_2_ at 300 °C for 2 h. The CuO content of both catalysts was 50%.

**Figure 7:**
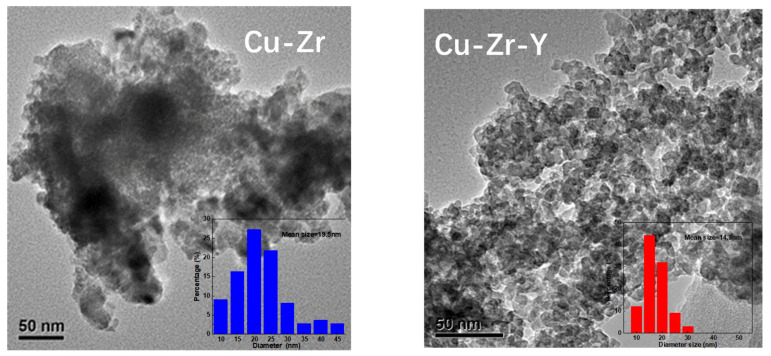
The TEM images and particle size distribution of the reduced Cu-Zr and Cu-Zr-Y catalysts.

For both catalysts, four peaks can be observed. The broad peak at 21° was attributed to ZrO_2_. This observation indicated that the support of the reduced catalyst was mainly present in the form of an amorphous phase. The three peaks at 43.2, 50.3, and 74.2° were attributed to metallic Cu [24] (PDF, 04-0836). There was no peak attributed to CuO, and no Cu_2_O can be detected, which suggested that Cu oxide was completely reduced to Cu^0^ during the reduction process. It was noted that the Cu^0^ peaks of Cu-Zr were much higher and stronger than those of Cu-Zr-Y. The weaker Cu^0^ peaks indicated that Cu-Zr-Y possessed better Cu metal dispersion than Cu-Zr. The TEM results, shown in Figure 7, further confirmed that the particle size of Cu metal over Cu-Zr-Y was smaller than that of Cu-Zr. For Cu-Zr, the mean size of the Cu particle was 19.6 nm, and there were some big particles with diameters bigger than 30 nm that were observed. For Cu-Zr-Y, the mean size of the Cu particle was 14.3 nm, and most of the particles possessed diameters in the range of 10 to 20 nm. Clearly, the addition of Y_2_O_3_ effectively increased the Cu^0^ dispersion. This was one possible reason why Cu-Zr-Y possessed better catalytic performance.

To study the effect of Y_2_O_3_ on the Cu dispersion, the specific surface areas of the Cu-Zr and Cu-Zr-Y catalysts were determined by N_2_ adsorption–desorption experiments. The results indicated that the Cu-Zr-Y catalyst possessed a much higher surface area than the Cu-Zr catalyst. The surface area of the former was 105.3 m^2^/g, while the latter was 62.0 m^2^/g. Generally, a high surface area favors good dispersion. One possible reason for the better Cu dispersion was that Y_2_O_3_ increased the catalyst-specific surface area.

**Figure 8:**
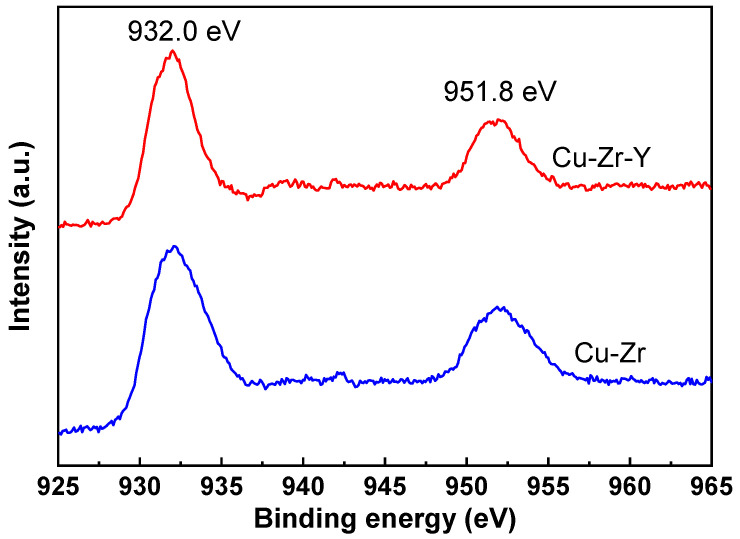
The XPS results of the reduced Cu-Zr and Cu-Zr-Y catalysts.

Figure 8 shows the XPS results of the Cu-Zr and Cu-Zr-Y catalysts reduced by H_2_ at 300 °C for 2 h. It can be seen that the two catalysts exhibited very similar XPS patterns, where two peaks at 932.0 and 951.8 eV were observed over both catalysts. It was reported that both Cu^0^ and Cu^+^ gave signals at ~932.0 and ~951.8 eV, and it is hard to discriminate Cu^0^ and Cu^+^ [25]. The XRD results, shown in Figure 6, suggested that the Cu species of the reduced Cu-Zr and Cu-Zr-Y was mainly present in the form of Cu^0^, and no CuO_2_ was detected. Therefore, we inferred that the peaks at 932.0 and 951.8 eV was mainly attributed to Cu^0^. The H_2_-TPR results in the following will further confirm this supposition.

**Figure 9:**
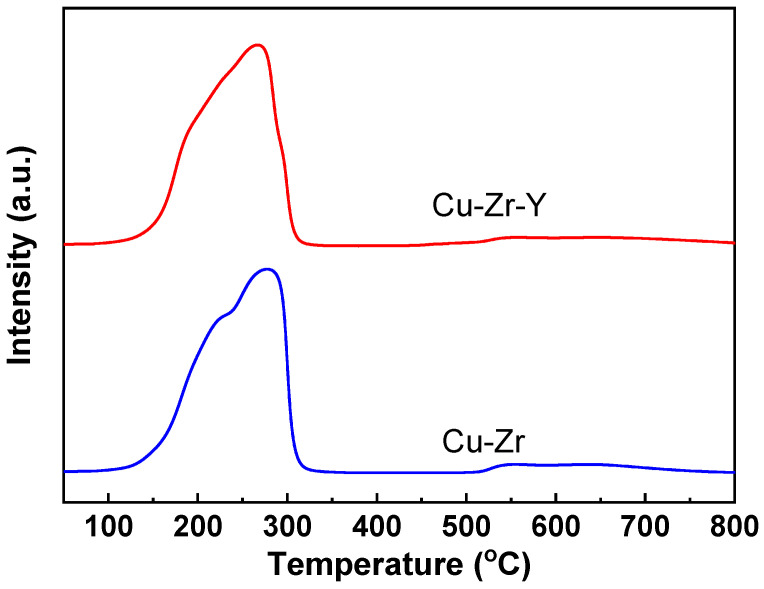
The H_2_-TPR results of the Cu-Zr and Cu-Zr-Y catalysts.

Figure 9 presents the H_2_-TPR results of Cu-Zr and Cu-Zr-Y. The Cu-Zr catalyst showed two reduction peaks, one at around 225 °C and another at around 280 °C. The lower temperature peak was attributed to the reduction of CuO to Cu_2_O and the higher temperature peak to the reduction of Cu_2_O to Cu^0^. Cu-Zr-Y also presented two reduction peaks, indicating the reduction process of CuO→Cu_2_O→Cu^0^. However, the reduction temperatures were shifted to lower temperatures (~205 and ~265 °C, respectively). Since the H_2_-TPR method is dynamic, it is believed that a temperature of about 250 °C is sufficient to reduce both catalysts. The H_2_-TPR results further confirmed the XRD and XPS results, where only Cu^0^ was detected over the Cu-Zr and Cu-Zr-Y reduced by H_2_ at 300 °C for 2 h. Although Cu-Zr-Y can be completely reduced at 250 °C, the catalyst reduced at 250 °C exhibited a poorer selectivity than the reduced at 300 °C. The possible reason for the lower selectivity was that the reduction at 250 °C resulted in the mismatch between Cu metal sites and acidic sites since both Cu metal sites and acidic sites are required for the dehydration of glycerol to acetol. It was reported that the reduction temperature of the well-distributed CuO species or small copper oxide clusters interacting weakly with the support were lower than large CuO particles or the aggregated CuO clusters [21]. The results of XRD and TEM implied that Cu-Zr-Y possessed better Cu dispersion than Cu-Zr. It is reasonable to think that the smaller Cu metal particle size resulted in the lower reduction temperature of Cu-Zr-Y.

**Figure 10:**
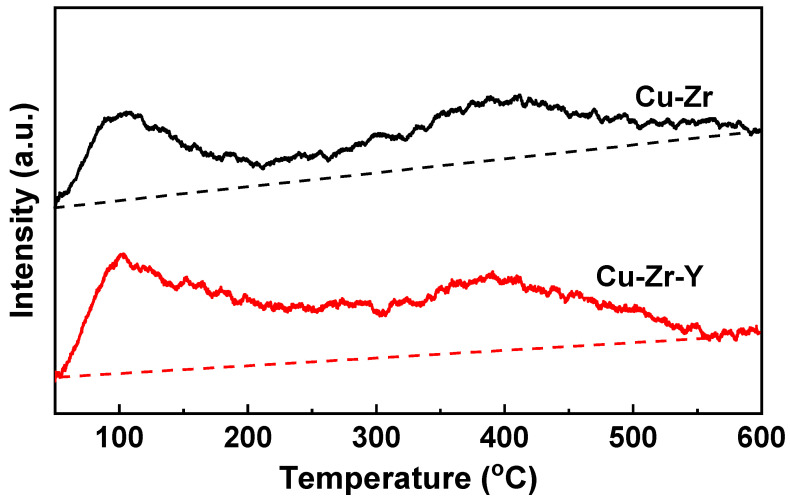
NH_3_-TPD profiles of Cu-Zr and Cu-Zr-Y.

It is well known that the acidity property of the catalyst strongly affects the catalytic performance for the dehydration of glycerol to acetol. NH_3_-TPD investigation was carried out, and the results are shown in Figure 10. Cu-Zr exhibited two NH_3_ desorption peaks, one at ~105 °C and another at ~395 °C. The former was attributed to the NH_3_ desorption of the weak acid sites while the latter to NH_3_ desorption of the strong acid sites. Cu-Zr-Y also exhibited two NH_3_ desorption peaks, however, the areas of the two peaks were bigger than the corresponding peaks of Cu-Zr. The bigger area indicated that the addition of Y_2_O_3_ increased the amount of acid sites. It was reported that increasing the copper oxide dispersion can develop the catalyst acidity and activity for dehydrate glycerol selectively to acetol [30,31]. Generally, the greater acidity the catalyst possesses, the better catalytic performance it exhibits [1,7]. Therefore, it is reasonable to think that the presence of Y_2_O_3_ facilitates the Cu dispersion, enhances the acidity, and thus promotes the selectivity for acetol production from glycerol.

**Figure 11:**
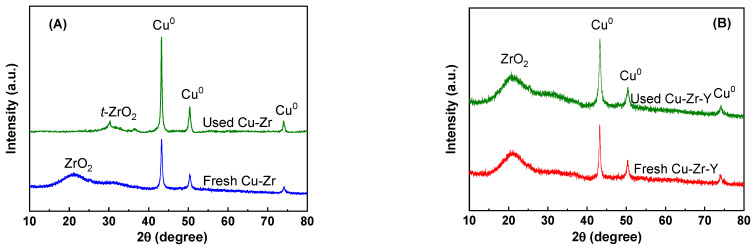
The XRD patterns of Cu-Zr (**A**) and Cu-Zr-Y (**B**) before and after reaction.

In order to further explore the promotional effect of Y_2_O_3_ for the stability, the used Cu-Zr and Cu-Zr-Y after the 30 h reaction were characterized by XRD. The results are shown in Figure 11. For the sake of comparison, the results of the reduced catalysts (by H_2_ at 300 °C for 4 h) were also included. As shown in Figure 11A, for the Cu-Zr catalyst, the XRD peak of the ZrO_2_ was shifted from 21.2 to 30.3° after the reaction, which suggested the formation of *t*-ZrO_2_. This result indicated that ZrO_2_ support was transformed from the amorphous phase to the *t*-ZrO_2_ phase during the reaction process. It also can be seen that the CuO^0^ peaks of the used catalyst were much sharper and higher than the reduced catalyst. This observation suggested that Cu^0^ particles were aggregated to a great extent during the reaction process. It can be seen from Figure 11B that the used Cu-Zr-Y catalyst exhibited a very similar XRD pattern to the reduced catalyst. This result indicated that the main structure of the catalysts, including the support and active metal, was retained during the reaction process.

**Figure 12:**
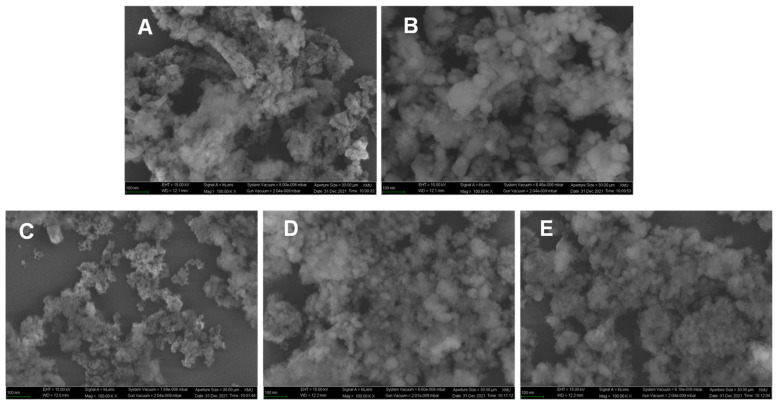
The SEM images of Cu-Zr and Cu-Zr-Y before and after reaction. (**A**): the reduced Cu-Zr; (**B**): the Cu-Zr after a 30 h reaction; (**C**): the reduced Cu-Zr-Y; (**D**,**E**): the Cu-Zr-Y after a 30 h and 250 h reaction.

Figure 12 presents the SEM images of Cu-Zr and Cu-Zr-Y before and after the reaction. For the reduced Cu-Zr and Cu-Zr-Y catalysts, they presented a cotton-like structure. No big particles can be observed. One possible reason for such morphology was that the amorphous phase ZrO_2_ resulted in such a structure. After the 30 h reaction, the morphology of Cu-Zr changed a lot. The cotton-like structure was transformed to a spherical structure, and the diameter of the sphere was in the range of 20 to 60 nm, indicating a sever aggregation. After the 30 h reaction, the morphology of Cu-Zr-Y also changed to some extent. Some of the catalyst was still present in the form of a cotton-like structure, while the other was transformed to a spherical structure. However, the diameter of the sphere was much smaller than that of the one used in Cu-Zr. This observation suggested that Cu-Zr-Y possessed a better ability against aggregation than Cu-Zr. It should be pointed that, even after the 250 h reaction, Cu-Zr-Y exhibited a very similar morphology with the one reaction at 30 h. The unchanged morphology further confirmed the excellent ability against aggregation.

For the dehydration of glycerol to acetol, both Cu metal sites and acidic sites are required for catalyzing the reaction. Generally, higher Cu dispersion and higher acidity amounts will result in higher activity and selectivity. Compared with Cu-Zr, Cu-Zr-Y possessed better Cu dispersion and higher acidity content, which ensured Cu-Zr-Y gave a better catalytic performance, especially for the selectivity. During the reaction process, Cu aggregation and support phase transformation maybe occur, which will decrease Cu sites and acidic sites and thus result in deactivation. The Cu aggregation and ZrO_2_ phase transformation from amorphous phase to *t*-ZrO_2_ phase was the main reason for the Cu-Zr deactivation. The presence of Y_2_O_3_ can effectively improve the ability of Cu metal against aggregation and inhibit support phase transformation under the reaction conditions, which greatly reduced the loss of Cu metal sites and acidic sites and thus enhanced the stability.

## 3. Materials and Methods

### 3.1. Materials

Cu(NO_3_)_2_·5H_2_O, Zr(NO_3_)_4_·5H_2_O, Y(NO_3_)_3_·6H_2_O and Na_2_CO_3_ were purchased from Sinopharm Chemical Reagent Co., Ltd. (Shanghai, China). Water used in all experiments was deionized water. All reagents were of analytical grade and used without further purification.

### 3.2. Preparation of Catalyst

The catalysts were prepared by a conventional coprecipitation method. First, the desired Cu(NO_3_)_2_·5H_2_O, Zr(NO_3_)_4_·5H_2_O, Y(NO_3_)_3_·6H_2_O was dissolved into deionized water. The obtained solution denoted solution A. The desired Na_2_CO_3_ was dissolved into deionized water to form solution B. Second, both solution A and solution B were dropwise added into a Pyrex flask under robust stirring. The temperature was kept at 80 °C, and the pH at about 7.5. After coprecipitation process, the suspension was continuously stirred for 30 min at 80 °C. Third, when temperature cooled down to room temperature, the mixture was filtered and washed with deionized water. Finally, the obtained solid was dried at 120 °C for 12 h, calcined at 400 °C for 4 h, and then obtained the powder catalysts. All the powder catalysts were formed to a size of 40–80 mesh for the evaluation of catalytic performance. For all catalysts, the mass ration of ZrO_2_:Y_2_O_3_ was 4:1. If not specially specified, the CuO content of Cu-Zr and Cu-Zr-Y was 50%.

### 3.3. Characterization of Catalysts

To obtain the powder XRD (Rigaku Ultima Ⅳ XRD) patterns, samples were scanned using CuKα radiation in the 2θ range of 10–80° at a rate of 10°/min. N_2_ adsorption–desorption experiments were performed using Micromeritics ASAP 2020, and the surface area were obtained by the Brunauer–Emmett–Teller (BET) equation. ICP-MS was used to determine the Cu content on an Agilent 4500 ICPMS. SEM (SUPRA 55) were used to observe the catalyst morphology. TEM (TECNAI F30) was used to analyze the size of Cu particles of the catalysts. XPS was used to analyze the Cu chemical structure of the catalysts on a PHI Quantum 2000 Scanning ESCA Microprobe (Physical Electronics, Chanhassen, MN, USA) using monochromatic Al Kα radiation (1846.6 eV) as the X-ray source. H_2_-TPR was used to study the reducibility of the catalyst and carried out on a Micromeritics AutoChem II 2920 instrument equipped with a TCD detector. NH_3_-TPD was used to study the acidity of the catalyst and performed in a home-made setup equipped with mass spectrometry.

### 3.4. Catalytic Performance

Catalytic tests were performed in a fixed bed microreactor under atmospheric pressure, using 2.0 g of catalyst. Prior to reaction, catalysts were first reduced in H_2_ (60 mL/min) at temperatures in the range 250 to 300 °C for 2 h. If not special specified, the reduction temperature was 300 °C. Then, an aqueous solution of glycerol at 70 wt.% was pumped at feed rates in the range of 0.64 to 1.92 mL/h, then vaporized and fed through the reactor in a down flow mode together with a gas (H_2_-5% and N_2_-95%) flow rate of 60 mL/min. The catalytic tests were carried out at temperature in the range of 180 to 260 °C for at least 24 h. The liquid products were collected in an ice–water–salt trap and analyzed by a gas chromatography equipped with a flame ionization detector (FID) and Rtx-Wax column (30 m 0.25 mm). For all testing, the yield to liquid products were higher than 96%, and no gaseous products was detected. Glycerol conversion, acetol selectivity, and 1,2 propanediol selectivity were calculated in the following way:

$$C_{\mathrm{Glycerol}}=\frac{Amount\;of\;glycerol\;consumed}{Amount\;of\;glycerol\;introded\;into\;the\;reactor}\times 100\%$$

$$S_{Acetol}=\frac{Amount\;of\;obtained\;acetol}{Amount\;of\;glycerol\;consumed}\times 100\%$$

$$S_{1,2propanediol}=\frac{Amount\;of\;obtained\;1,2\;propanediol}{Amount\;of\;glycerol\;consumed}\times 100\%$$

## 4. Conclusions

The dehydration of glycerol to acetol was studied over Cu-Zr and Cu-Zr-Y catalysts. The results indicated that Cu-Zr-Y was much more effective and stable than Cu-Zr. The Cu-Zr-Y with 50% CuO possessed high activity, selectivity, and stability. The high glycerol conversion (~100%) and acetol selectivity (~85%) remained unchanged during a 250 h catalytic reaction. In contrast, Cu-Zr with 50% CuO showed a lower selectivity and suffered deactivation. The characterization results of the reduced (or calcined) catalysts suggested that the presence of Y_2_O_3_ increased the surface area, improved Cu dispersion, and enhanced acidity content, which was mainly responsible for the high activity and selectivity. The characterization results of the used catalysts indicated that the addition of Y_2_O_3_ stabilized the main structure of the catalyst, suppressed Cu from aggregation, and kept support from phase transformation during the reaction, resulting in the good stability. Overall, this work not only presents an efficient catalyst with potential applications for the dehydration of glycerol to acetol, but also provides a strategy to rationally design active and stable Cu/Zr catalysts by using an oxide promoter.

## Figures and Tables

**Table 1:** The effect of CuO content on the activity and selectivity of the Cu-Zr-Y catalyst.

CuO Content (%)	Conversion (%)	Selectivity (%)	
		Acetol	1,2-Propanediol
45	99.6	81.7	6.8
50	99.8	85.0	7.2
55	99.1	84.3	6.5
60	99.5	83.1	7.6

Conditions: temperature = 220 °C, Time = 30 h, LHSV = 0.45 h^−1^.

**Table 2:** The effect of reduction temperature on the catalytic performance of Cu-Zr-Y.

Temperature (°C)	Conversion (%)	Selectivity (%)
250	99.1	78.5
300	99.5	85.4
350	99.7	82.7

Conditions: temperature = 220 °C, Time = 30 h, LHSV = 0.45 h^−1^.

## Data Availability

The data presented in this study are available on request from the corresponding author.
